# Overcoming Chemoresistance: Altering pH of Cellular Compartments by Chloroquine and Hydroxychloroquine

**DOI:** 10.3389/fcell.2021.627639

**Published:** 2021-02-09

**Authors:** Peter W. Halcrow, Jonathan D. Geiger, Xuesong Chen

**Affiliations:** Department of Biomedical Sciences, School of Medicine and Health Sciences, University of North Dakota, Grand Forks, ND, United States

**Keywords:** chloroquine, hydroxychloroquine, chemoresistance, cytosolic pH, endolysosome pH, Golgi pH

## Abstract

Resistance to the anti-cancer effects of chemotherapeutic agents (chemoresistance) is a major issue for people living with cancer and their providers. A diverse set of cellular and inter-organellar signaling changes have been implicated in chemoresistance, but it is still unclear what processes lead to chemoresistance and effective strategies to overcome chemoresistance are lacking. The anti-malaria drugs, chloroquine (CQ) and its derivative hydroxychloroquine (HCQ) are being used for the treatment of various cancers and CQ and HCQ are used in combination with chemotherapeutic drugs to enhance their anti-cancer effects. The widely accepted anti-cancer effect of CQ and HCQ is their ability to inhibit autophagic flux. As diprotic weak bases, CQ and HCQ preferentially accumulate in acidic organelles and neutralize their luminal pH. In addition, CQ and HCQ acidify the cytosolic and extracellular environments; processes implicated in tumorigenesis and cancer. Thus, the anti-cancer effects of CQ and HCQ extend beyond autophagy inhibition. The present review summarizes effects of CQ, HCQ and proton pump inhibitors on pH of various cellular compartments and discuss potential mechanisms underlying their pH-dependent anti-cancer effects. The mechanisms considered here include their ability to de-acidify lysosomes and inhibit autophagosome lysosome fusion, to de-acidify Golgi apparatus and secretory vesicles thus affecting secretion, and to acidify cytoplasm thus disturbing aerobic metabolism. Further, we review the ability of these agents to prevent chemotherapeutic drugs from accumulating in acidic organelles and altering their cytosolic concentrations.

## Introduction

Chemoresistance is a process by which cancer cells evade chemotherapeutic drug-induced cell death. Overcoming chemoresistance represents a major challenge to people living with cancer and their caregivers. A diverse set of cellular and inter-organellar signaling pathways that cancer cells use to promote their own survival and avoid chemotherapeutic agent-induced apoptosis has been described (Senthebane et al., 2017; Zheng, 2017), but the underlying processes responsible for chemoresistance remain unclear and effective strategies to overcome chemoresistance are still lacking.

The progression of cancer and it’s resistance to chemotherapy are well-known to be linked to tumor microenvironments (Gerweck et al., 1999). The acid-base balance of microenvironments is of particular importance in that pH is critical for essential biological processes including protein folding, enzyme activity, vesicle trafficking, and inter-organellar signaling. Cancer cell survival is also conditioned on acid-base balance, and a hallmark of tumor microenvironments is the acidic extracellular matrix that is directly linked to a more alkaline cytosolic microenvironment (Swietach et al., 2014). Such acid-base microenvironment adaptations profoundly alter the metabolism of cancer cells that support their special energetic needs. Furthermore, the acid-base microenvironment adaptation occurs in subcellular organelles including endolysosomes (Higgins, 2007; Appelqvist et al., 2013; Sui et al., 2013) and Golgi (Rivinoja et al., 2006, 2009); changes known to contributes to the survival of cancer cells, malignant progression, and the development of chemoresistance.

The anti-malaria drugs, chloroquine (CQ) and its derivative hydroxychloroquine (HCQ), have been used for in the treatment of various cancers often in combination with chemotherapeutic drugs due to their ability to enhance the efficacy of tumor cell killing (Briceño et al., 2003; Boone et al., 2015; Xu et al., 2018). The widely accepted mechanism by which CQ and HCQ exert their anti-cancer effects is their ability to inhibit autophagic flux (Amaravadi et al., 2011; Ye et al., 2016; Mauthe et al., 2018). As diprotic weak bases, CQ and HCQ preferentially accumulate in acidic organelles (endolysosomes and Golgi) and neutralize their luminal pH. However, simultaneously, CQ and HCQ acidify the cytosol and de-acidify the extracellular environment (Xia et al., 2018; Halcrow et al., 2019a,b). Thus, the anti-cancer effects of CQ and HCQ might extend beyond autophagy inhibition.

To strengthen the application and possible use of CQ and HCQ as adjuvant therapies to overcome chemoresistance, it is important to understand better the effects of CQ and HCQ on pH, inter-organellar interactions, and other cellular processes from a subcellular perspective. Accordingly, the present review will summarize the microenvironment (pH) adaptation of cancer cells and discuss potential mechanisms underlying acid/base-dependent anti-cancer effects of CQ and HCQ. These include their ability to de-acidify lysosomes and inhibit autophagosome lysosome fusion, to de-acidify Golgi apparatus and secretory vesicles thus affecting secretion, and to acidify the cytoplasm thus disturbing aerobic metabolism. Further, we will discuss their ability to prevent chemotherapeutic drugs from accumulating in acidic organelles thus increasing cytosolic concentrations of anti-cancer drugs.

## Tumor Microenvironment

### Extracellular Microenvironment

Observed by Warburg and Pasteur in aerobic glycolysis and hypoxia, the physiological pH of the extracellular microenvironment of normal tissue (pH 7.4) becomes acidic (pH 6.5) in tumor microenvironments due to the excretion of lactic acid (Bailey et al., 2012). This acidification of the extracellular microenvironments around tumors not only enhances the activity of proteinases such as metalloproteinase and released lysosomal enzymes, but also affects transcription via activation of signal transduction across the plasma membrane, and thus augments the malignancy and aggression of cancer (Schornack and Gillies, 2003; Gatenby et al., 2006; Moellering et al., 2008; Hashim et al., 2011; Kato et al., 2013). Acidification of the extracellular microenvironment also creates an obstacle of the proton-gradient at the plasma membrane that reduces the uptake of chemotherapeutic agents such as mitoxantrone and topotecan, and thus generates tumors resistant to chemotherapy and immunotherapy (Thews et al., 2006; Kato et al., 2013; Pilon-Thomas et al., 2016).

### Cytosol

The cytosolic pH of normal cells (pH 6.99–7.2) becomes more alkaline (pH 7.12–7.65) in cancer cells (Oberhaensli et al., 1986; Ng et al., 1989; Vaupel et al., 1989; Baghdadi, 2017). The acidic nature of the extracellular environment and a more alkaline cytosolic microenvironment in cancer cells generates a proton-gradient across the plasma membrane that becomes a source of proton-motive energy, and cancer cells use this energy to synthesize phosphate bonds (pyrophosphate) from free phosphate and generate ATP independently of glycolysis and mitochondrial ATP. This cellular phenomenon of the proton gradient across plasma membranes further stimulates the energy needs of cancer cells and stimulates tumor cell proliferation (Dhar et al., 2015).

In cancer cells, the metabolism mechanisms switch to glycolysis for ATP production; a process first described by Otto Warburg in 1924 that became known as the “Warburg effect.” Regardless of the availability of O_2_, cancer cells increasingly convert glucose into lactate and utilize the glucose metabolites to accelerate cell proliferation at the expense of generating two molecules of ATP per molecule of glucose. Tumors, which demand high levels of energy, make up for this ATP loss by going through glycolysis much faster and more often than do normal cells. Indeed, Warburg hypothesized that tumor formation originated due to irreversible damage to mitochondrial oxidative phosphorylation. Under normal oxygen tension conditions, Warburg observed normal cells produced most of their energy from mitochondrial respiration while over 50% of the energy generated in cancer cells originated in the cytosol from glycolysis and the remainder from mitochondrial respiration. However, the reliance of cancer cells on glycolysis for most of their energy is not due to lack of oxygen, because even in the presence of adequate oxygenation cancer cells still operate mainly via glycolysis (Fadaka et al., 2017).

An increase in cytosolic pH (alkalization) can shift metabolism from oxidative phosphorylation to glycolysis under aerobic conditions in cancer cells (Reshkin et al., 2000, 2013; Nagata et al., 2011). The Warburg effect can be explained by an increase in cytosolic pH in cancer cells. The alkalinization of the cytosolic microenvironment initiates aerobic glycolysis (Reshkin et al., 2000) and hydrogen ions may be one of the most significant factors responsible for determining how cancer cells obtain energy. Even in the presence of adequate oxygen, an alkaline cytosolic pH drives aerobic glycolysis, and an acidic intracellular pH drives oxidative phosphorylation (OXPHOS) (Harguindey et al., 2005; López-Lázaro, 2010). Thus, OXPHOS and glycolysis are oppositely pH sensitive. Furthermore, an alkaline cytosolic pH activates the key glycolytic enzyme phosphofructokinase and inhibits gluconeogenesis (Trivedi and Danforth, 1966; Harguindey et al., 1979). In addition, an alkaline cytosolic pH inhibits OXPHOS and pyruvate entry into the Krebs cycle (Relman, 1972). Therefore, damaged mitochondrial respiration is not the primary cause of cancer, as Warburg suggested, but the main cause of aerobic glycolysis in cancer cells appears to be a disruption of the acid-base homeostasis within cells (Alfarouk et al., 2014). The Na^+^/H^+^ exchanger isoform 1 (NHE1) proton transporter is known to be specifically involved in cellular acid-base balance in cancer cells, where it contributes to cytosolic pH homeostasis, cell transformation, proliferation, tumor growth, invasion, activation of the metastatic process, and resistance to chemotherapy (Cardone et al., 2005; Stock et al., 2008; Reshkin et al., 2013). This helps explain the vast literature on targeting NHE1 in cancer through the use of so-called proton pump inhibitors.

### Nucleus

Equivalent to the cytosolic microenvironment, the nuclear microenvironment in normal cells is slightly more alkaline (pH 7.55–7.88) than that of the cytosol (Cody et al., 1993; Seksek and Bolard, 1996; Masuda et al., 1998). However, little is known about how nuclear pH affects nuclear biology. Given the inherent affinity of nucleic acids and nuclear proteins for protons, it is predicated that nuclear pH alteration is likely to influence the activity of transcription factors and thus DNA replication (Hulikova and Swietach, 2016). Furthermore, chemotherapeutic drugs are designed to often work by targeting DNA processes and rapidly proliferating cells. The nucleus represents a major site where most chemotherapeutic compounds are likely to be effective in preventing DNA replication (Patel and Patel, 2012). Altered nuclear pH is also likely to alter the levels of nucleus-targeting chemotherapeutic compounds and affects their effectiveness in preventing DNA replication for cancer repression. However, presently unclear is the extent to which nuclear pH is changed in cancer cells.

### Endolysosomes

Endosomes and lysosomes (endolysosomes) represent a dynamic system of organelles exhibiting morphological and functional heterogeneity as well as complex interactions with other organelles. A hallmark of the endolysosome system is their acidic luminal pH (Matsuo et al., 2004; Huotari and Helenius, 2011; Mindell, 2012; Mcguire et al., 2017; Prasad and Rao, 2018), where early endosomes have a luminal pH of 6.0–6.6, late endosomes have a luminal pH of ∼5.0, and the lumen of lysosomes is most acidic with a pH as low as ∼4.5. The acidic environment is critical for the activity of up to 60 different pH sensitive hydrolytic enzymes including proteases, lipases and nucleases (De Duve, 2005). Although no direct measurements of alterations in endolysosome pH of cancer cells has been reported, changes in endolysosome volume and subcellular localizations have been reported during oncogenic transformation (Kirkegaard and Jaattela, 2009; Kallunki et al., 2013; Fennelly and Amaravadi, 2017), and such morphological changes indicate a functional adaption of endolysosomes including alterations in endolysosome pH, which occurs in cancer biology.

Classically known as garbage processing centers, endolysosomes are responsible for the degradation of extracellular macromolecules and membrane components as well as long-lived intracellular proteins and obsolete organelles. Up-taken into endosomes via endocytosis, extracellular macromolecules and membrane components can either be trafficked through early endosomes to recycling endosomes, which mediates receptor recycling to the plasma membrane or Golgi apparatus, or can even transition to late endosomes and fusion with lysosomes (Bright et al., 2005; De Duve, 2005; Luzio et al., 2007; Huotari and Helenius, 2011). The acidic pH of lysosomes and their pH-sensitive hydrolases are critical for the degradation of cytosolic proteins and organelles via the formation of autophagosomes, which are then fused with late endosomes/lysosomes; the so-called “self-eating” or autophagy, which is essential for cell homeostasis and development (Ohsumi, 2014; Spiering, 2019).

During autophagy, engulfed cytosolic macromolecules and organelles are broken down into building blocks used for metabolism and to produce energy and build new proteins, lipids, sugars, and amino acids. As such, under starvation conditions, autophagy becomes an internal source of nutrients and energy, promoting survival under harsh conditions as it fuels metabolism for cells under stress. It is known that harsh conditions, such as hypoxia, nutrient deprivation, and oxidative stress, exist in tumor microenvironments, thus, cancer cells exploit autophagy to survive. The number of autophagosomes is increased in tumors under hypoxic regions, and knocking out autophagy-regulating genes results in tumor cell death; thus, cancer cells exploit autophagy to survive (Mathew et al., 2007; Cheng et al., 2013). To avoid cell death, tumor cells can reprogram themselves metabolically in order to engage autophagy as a source of energy and survive (Deberardinis et al., 2008; Lozy and Karantza, 2012). In addition to the classical degradation role, endolysosomes control a variety of essential physiological functions including antigen processing, membrane repair, nutrient sensing, and ion homeostasis (Christensen et al., 2002; Casey et al., 2010; Xu and Ren, 2015; Pu et al., 2016); all of which can contribute to cancer progression.

Endolysosomes also play an important role in chemoresistance (Zhitomirsky and Assaraf, 2016; Geisslinger et al., 2020; Hrabeta et al., 2020). The acidic environment of endolysosomes facilitates luminal accumulation of lipophilic, weakly basic chemotherapeutic drugs. Hydrophobic weak-base chemotherapeutic drugs are predominately accumulated in endolysosomes via passive diffusion along the pH gradient or are actively transported across membranes into the endolysosome lumen by P-glycoproteins embedded in endolysosome membranes (Yamagishi et al., 2013; Al-Akra et al., 2018). Upon entering endolysosome lumen, weak-base chemotherapeutic drugs undergo protonation and are trapped in endolysosomes. Once trapped inside endolysosome, weak-base chemotherapeutic drugs can no longer reach the cytosolic compartment and the nucleus to exert their anticancer effects; processes linked to chemoresistance (Zhitomirsky and Assaraf, 2017). Such trapped weak-base chemotherapeutic drugs can directly alter endolysosome structure and function, such as alteration in endolysosome pH and influence endolysosome-dependent signaling that results in enhanced endolysosome biogenesis (Fassl et al., 2020). Alternatively, trapped weak-base chemotherapeutic drugs can be eliminated from the cell via an increase in the lysosomal exocytosis cellular process, which is known to be directly due to these drugs themselves (Hrabeta et al., 2020).

### Golgi Apparatus and Secretory Vesicles

Golgi apparatus are mildly acidic with luminal pH values ranging from 6.7 at *cis*-Golgi to 6.0 at trans-Golgi (Kellokumpu, 2019). Secretory vesicles are more acidic with luminal pH ranging from 5.2 to 5.7 (Demaurex, 2002; Paroutis et al., 2004). The acidic environments in Golgi are critical for processing, sorting, and trafficking of proteins and lipids destined for secretion, membranes, and organelles. Golgi pH in malignant cells is more alkaline than non-malignant cells (Rivinoja et al., 2006, 2009) and anion channels regulate Golgi pH (Maeda et al., 2008; Maeda and Kinoshita, 2010). Alkalization of Golgi lumen results in defects in post-translational modifications and processing of secreted proteins; glycosylation is pH-sensitive (Axelsson et al., 2001; Maeda et al., 2008; Maeda and Kinoshita, 2010) and a slight increase in pH results in decreased glycosylation (Rivinoja et al., 2006). Altered glycosylation is a hallmark of cancer phenotypes (Meezan et al., 1969) and promotes tumorigenesis and metastasis (Hakomori, 2002; Häuselmann and Borsig, 2014; Peixoto et al., 2016). The loss of the O-glycosyltransferase heteromers or enzyme mis-localization are thought to be the two main reasons for the pH-dependent glycosylation changes seen in cancer cells (Jass et al., 1994; Kuhns et al., 1995; Hakomori, 2002; Kellokumpu, 2019). With respect to the sorting and trafficking of proteins and lipids, alkalization of Golgi can impair anterograde transport from Golgi to secretary vesicles (Chanat and Huttner, 1991), retrograde transport from Golgi back to the endoplasmic reticulum (Palokangas et al., 1998), the delivery of lysosomal hydrolases to lysosomes via mannose-6-phosphate receptor (Ghosh et al., 2003; Kokkonen et al., 2004), the integrity of Golgi itself (Linstedt et al., 1997; Puri et al., 2002), and the sorting and proteolytic maturation of prohormones in secretory granules (Schmidt and Moore, 1995); all of which contribute to the malignant nature of cancer cells.

## Chloroquine De-Acidifies Acidic Organelles and Acidifies the Cytosol

As diprotic weak-base drugs, CQ (pK_a1_ = 8.1, pK_a2_ = 10.2) and HCQ (pK_a1_ = 8.3, pK_a2_ = 9.7) are present in protonated and unprotonated forms. Unprotonated forms of CQ and HCQ can diffuse freely across membranes into the lumen of acidic endolysosomes and Golgi. Once protonated, CQ and HCQ become trapped in the lumen of these acidic organelles (Martin et al., 2009; Solomon and Lee, 2009). The driving force for intra-luminal accumulation of CQ and HCQ is proportional to the square of the hydrogen ion gradient. Thus, the driving force is much larger than that of a monoprotic weak base like ammonia chloride, which is proportional to the hydrogen ion gradient (De Duve et al., 1974; Roos and Boron, 1981). Thus, CQ and HCQ are preferentially concentrated in and neutralize the acidic pH of organelles including endolysosomes (Homewood et al., 1972; Schlesinger et al., 1988; Dunmore et al., 2013; Khan et al., 2019) and Golgi (Rivinoja et al., 2006, 2009; Kellokumpu, 2019). In addition, chloroquine acidifies the cytosol by 0.2–0.4 pH units within 1 h of drug exposure (Xia et al., 2018). Therefore, CQ and HCQ can de-acidify acidic organelles as well as acidify the cytoplasm; effects linked to overcoming chemoresistance.

## CQ-Induced Inhibition of Autophagic Flux Enhances the Efficacy of Chemotherapeutic Drugs

Tumor cells can be reprogrammed metabolically in order to engage autophagy as a source of energy and survival (Deberardinis et al., 2008; Lozy and Karantza, 2012). CQ and HCQ can directly inhibit the formation of autolysosomes (Boya et al., 2005; Jahreiss et al., 2008; Klionsky et al., 2016). More specifically, CQ and HCQ increase the pH of lysosomes and inhibit the activity of the hydrolase enzymes, which inhibits the fusion of autophagosomes with lysosomes and thus blocks the degradation of engulfed macromolecules and damaged organelles (Amaravadi et al., 2011; Ye et al., 2016; Mauthe et al., 2018). CQ treatment alone has been shown to inhibit cancer cell growth, as well as enhance apoptosis through the inhibition of autophagic flux (Ye et al., 2016). Furthermore, CQ-induced inhibition of autophagic flux can enhance the apoptotic efficacy of anticancer drugs (Cheng et al., 2013). As a pro-survival mechanism, autophagy promotes cancer cell fitness under stressful conditions, as well as acts as a protective mechanism for tumor cells against chemotherapy and promotes drug resistance (White and Dipaola, 2009; Al-Akra et al., 2019). Thus, CQ-induced inhibition of autophagic flux could enhance the cytotoxicity of temozolomide and increase radiosensitivity in glioblastoma (Taylor et al., 2018). It also has been shown that autophagy activation contributes to cancer cells resistant to cisplatin due to aquaporin 3 (AQP3) over-expression, and CQ-induced autophagy inhibition reverses the chemoresistance caused by AQP3 over-expression (Dong et al., 2016).

## CQ-Induced De-Acidification Prevents Endolysosome Accumulation of Chemotherapeutic Drugs and Increases Their Concentrations in the Cytosol and Nucleus

**FIGURE 1:**
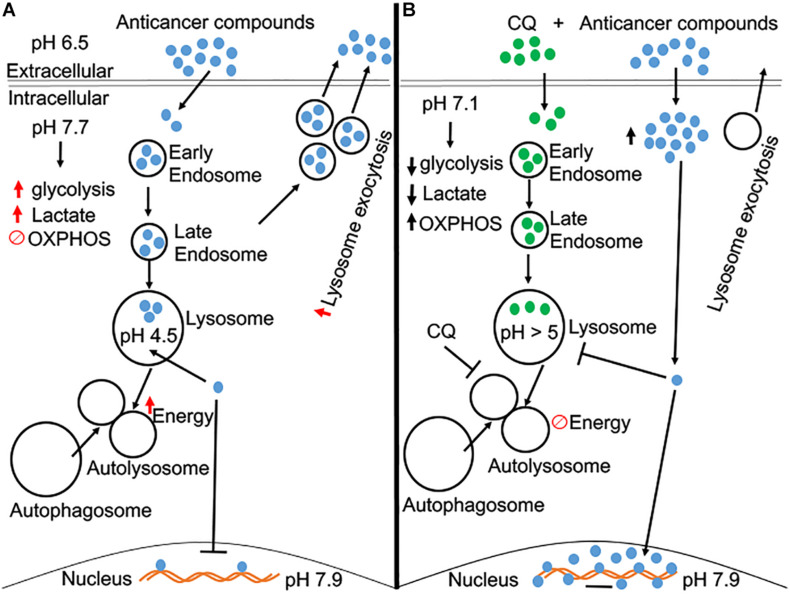
A schematic diagram of CQ pre-treated in overcoming chemoresistance. **(A)** Beside acidic extracellular microenvironment, tumor cells have a slightly alkaline cytosolic microenvironment, which leads to an increase in aerobic glycolysis. Anti-cancer compounds (many of them are weak bases) tend to accumulate in acidic organelles, like endolysosomes, and increase exocytosis of these compounds back into the extracellular matrix. Thus, the designated effect of these anti-cancer compounds is futile, and the cell becomes resistant to these weak-base anti-cancer compounds. **(B)** Pre-treatment of CQ acidifies the cytosolic microenvironment and may reverse the increase of aerobic glycolysis as occurs in cancer cells. Pre-treatment of CQ de-acidifies endolysosomes and inhibits autophagic flux. CQ-induced endolysosome de-acidification prevents the accumulation of anti-cancer compounds in endolysosomes and increases their concentration in the cytosol and nucleus, the sites where anti-cancer compounds exert their anti-cancer effects.

Hydrophobic weak-base chemotherapeutic drugs accumulate in endolysosomes (Jansen et al., 1999; Kaufmann and Krise, 2007; Adar et al., 2012; Zhitomirsky and Assaraf, 2016), de-acidify endolysosomes (Kazmi et al., 2013), promote increased lysosomal exocytosis (Zhitomirsky and Assaraf, 2017), and enhance endolysosome biogenesis (Zhitomirsky and Assaraf, 2015); all of which contributes to the development of chemoresistance (Jansen et al., 1999; Gotink et al., 2011; Zhitomirsky and Assaraf, 2015). These cellular phenomena between weak-base chemotherapeutic drugs and endolysosomes brings to the forefront the critical role that endolysosomes play in the development of chemoresistance in cancer cells (Figure 1A).

As diprotic weak-base drugs, CQ and HCQ are preferentially concentrated in endolysosomes (Morissette et al., 2004; Solomon and Lee, 2009; Marceau et al., 2012), where they neutralize the acidic luminal pH (Schlesinger et al., 1988; Khan et al., 2019), induce enlargement of endolysosomes (Yoon et al., 2010; Mauthe et al., 2018), alter the positioning of endolysosomes from perinuclear to the periphery of cells (Johnson et al., 2016; Datta et al., 2019), and lead to lysosome membrane permeabilization (Circu et al., 2017; Sironi et al., 2019; Khan et al., 2020). Thus, CQ-induced endolysosome de-acidification alone could lead to cytotoxic effects in cancer cells. Also, CQ-induced endolysosome de-acidification could result in accumulation and aggregation of un-degraded substrates and atypical cleavages that lead to the generation of toxic intermediates (Caporaso et al., 1992; Hui et al., 2019). Furthermore, CQ-induced lysosomal membrane permeabilization leads to the translocation of lysosomal contents (e.g., cathepsins) to the cytoplasm, which results in the induction of mitochondria damage and cell death (Boya, 2012; Wang et al., 2018).

Interestingly, HCQ pretreatment at a low concentration (5 μM) enhances the sensitivity of tumor cells to doxorubicin (Li et al., 2018). At this low concentration, HCQ caused no cytotoxicity, which indicates that the diprotic weak-base HCQ can function as a sensitizing agent to enhance the intended purpose of chemotherapeutic agents without killing cells directly. Moreover, HCQ pre-treatment enhanced the effects of other chemotherapeutic agents, including paclitaxel, mitomycin C, and cisplatin, in inhibiting tumor proliferation. Also, increased entry of doxorubicin across the plasma membrane into the cytoplasm in the A549 cancer cell line was observed with HCQ pre-treatment (Li et al., 2018). Most importantly in this same study, the accumulation of doxorubicin in lysosomes was blocked when cells were pre-treated with HCQ. These findings indicate that HCQ and CQ enhance the sensitivity of cancer cells to chemotherapeutic drugs by increasing the concentration of chemotherapeutic compounds across the plasma membrane into the cytoplasm, preventing accumulation of chemotherapeutic drugs in lysosomes, and thus increase their concentrations in cytosolic and nuclear compartments; the sites where chemotherapeutic effects occur (Figure 1B; Liu et al., 2014; Rebecca and Amaravadi, 2016; Ruiz et al., 2016; Tang et al., 2016; Yan et al., 2018).

## CQ-Induced Cytosolic Acidification Disturbs Aerobic Metabolism and Replication

Hydrogen ions are significant factors in determining how cancer cells obtain energy. The slight cytosolic alkalization, as occurs in cancer cells, can initiate aerobic glycolysis in cancer cells (Reshkin et al., 2000, 2013; Nagata et al., 2011), and thus contribute to the Warburg effect and profoundly affecting cancer cell metabolism. Reversing the pH gradient hallmark of cancer metabolism from the alkaline cytosolic pH back towards a slightly acidic pH could disable cell proliferation and tumor growth (Persi et al., 2018). As such, the ability of CQ to acidify the cytosol and reverse the slightly alkaline cytosolic pH, as present in cancer cells, back to physiological levels could help overcome chemoresistance, which represents a novel target of interest. It is currently not clear how CQ acidifies the cytosolic microenvironment.

## CQ-Induced Golgi De-Acidification and Chemoresistance

Golgi luminal pH is slightly increased in cancer cells, and such change in Golgi luminal pH can alter glycosylation, a hallmark of cancer phenotypes (Meezan et al., 1969). As diprotic weak-base drugs, CQ and HCQ are also concentrated in and neutralize the pH of acidic Golgi (Kellokumpu, 2019), and induce marked dilatation of the Golgi cisternae (Thorens and Vassalli, 1986). Little is known about how CQ affects the progression of a cancer cell that is already exhibiting an elevated luminal pH of Golgi. There is evidence indicating that CQ not only results in deficits in glycosylation (Rivinoja et al., 2006), but also leads to deficits in the formation of functional transport vesicles and impaired ability of budding vesicles to pinch off and form functional transport vesicles (Oda and Ikehara, 1985; Hiebsch et al., 1991). Such effects of CQ could potentially disturb the progression of cancer cells, which warrants further investigation. Furthermore, CQ-induced Golgi de-acidification can also change distribution patterns of mannose-6-phosphate receptors and decrease the delivery of lysosomal enzymes into lysosomes (Brown et al., 1984), which could, in another way, inhibit autophagic flux and enhance the apoptotic efficacy of chemotherapeutic drugs.

## CQ-Induced De-Acidification of Acidic Organelles and Immunomodulatory Properties

CQ and HCQ also exhibit immunomodulatory properties and are used clinically for the treatment of rheumatoid arthritis, systemic lupus erythematosus, and other inflammatory rheumatic diseases (Gotoff, 1968; Panayi et al., 1973; Schrezenmeier and Dorner, 2020). Although not fully understood, CQ- and HCQ-induced de-acidification of acidic organelles, including endolysosomes and Golgi, in immune cells could, at least in part, contribute to their immunomodulatory effects. Given that lysosomal degradation of endocytosed or autophagocytosed proteins plays an important role in antigen processing and MHC class II presentation (Lotteau et al., 1990; Munz, 2016), CQ- and HCQ-induced endolysosome de-acidification could inhibit MHC class II expression, antigen presentation and immune activation (Belizaire and Unanue, 2009). In addition, activation of toll-like receptor (TLR) signaling, and production of pro-inflammatory cytokines has been linked to binding of RNA and DNA to TLR7 and TLR9 in endosomes (Ewald et al., 2008). It is likely that CQ- and HCQ-induced endolysosome de-acidification could interfere with the binding of RNA and DNA to TLR7 and TLR9, and thus inhibit TLR signaling and the production of pro-inflammatory cytokines (Kuznik et al., 2011; Schrezenmeier and Dorner, 2020). Furthermore, CQ- and HCQ-induced de-acidification of endolysosomes and Golgi could contribute to their inhibitory effects on the release of various pro-inflammatory cytokines such as IL-1, IFNα, and TNFα (Savarino et al., 2003; Jang et al., 2006). For example, de-acidifying Golgi by CQ and HCQ blocked the conversion of pro-TNFα to its soluble mature form in Golgi (Jeong and Jue, 1997; Jang et al., 2006). CQ- and HCQ-induced de-acidification of endolysosomes and Golgi reduced the secretion of pro-inflammatory cytokines such as TNF-α, IL-1β, and IL-6 (Savarino et al., 2003; Jang et al., 2006); both Golgi (Murray and Stow, 2014) and endolysosomes (Carta et al., 2013; Zhang et al., 2015) are involved in cytokine release. Such anti-inflammatory effects may exacerbate the progression of cancer. On the other hand, CQ-induced lysosome de-acidification and amelioration of the tumor microenvironment has been implicated in its immunomodulatory effect, in which CQ mediates its antitumor efficacy via resetting tumor-associated macrophage from the M2 to the M1 phenotype (Chen et al., 2018; Li et al., 2018).

## CQ and HCQ Efficacy Based on Tumor Status

Effectiveness of CQ and HCQ in overcoming chemoresistance is also affected by specific protein expression and autophagic status of various tumors, which needs to be considered in drug-assisted therapy. As discussed, there has been much interest in targeting autophagy in cancer therapy based on preclinical findings (Guo et al., 2011; Yang et al., 2011). Even though CQ is under evaluation in clinical trials to target autophagy in cancer therapy, a current study demonstrated in oncogenic KRAS-dependent tumors that CQ was synergistic with tyrosine kinase inhibitors but not with ATG7 deficiency. These findings indicate that inhibition of autophagy may not be a relevant mechanism of action for CQ in certain cancer types (Eng et al., 2016). Furthermore, certain cancer types like acute myeloid leukemia (AML) render autophagy dispensable such that CQ-based treatment is ineffective in vivo due to limited drug efficacy in the blood (Chen et al., 2017). Thus, autophagic status of tumor cells may alter the effectiveness of CQ and HCQ. In addition, activation of the p53 pathway is a crucial strategy in targeting certain cancer types like glioblastoma, and CQ alone activates the p53 pathway and suppresses growth of glioma cells as well as metastatic tumor growth (Kim et al., 2010; Burikhanov et al., 2017). Adversely, HCQ accelerates tumor formation in oncogenic KRAS-dependent tumors that are p53 deficient (Rosenfeldt et al., 2013). Thus, tumor specific protein expression may alter the effectiveness of CQ and HCQ.

## Lysosome Membrane Permeability and Chemoresistance

An interesting and developing mechanism in chemoresistant therapy is lysosomal membrane permeability (LMP). Chemotherapeutic drug deposition into lysosomes generates chemoresistant cancer cells. Studies demonstrate that uptake of LMP inducers leads to cell death through ROS that disintegrate the membrane of lysosomes, thus, releasing entrapped chemotherapy agents to localize to the nucleus and induce cytotoxic effects (Seebacher et al., 2016). CQ enhances the production of endogenous nitric oxide that inhibits the activity of p-glycoprotein, which then localizes chemotherapy agents to the cytosol and nucleus and promotes cytotoxic effects in hepatic cancer (Li et al., 2017; Salaroglio et al., 2018). Furthermore, CQ with gemcitabine increases LMP and causes cathepsins to be released into the cytoplasm, which is associated with apoptosis (Bjørkøy et al., 2009; Fu et al., 2018; Sharapova et al., 2018).

## Adverse Effects of CQ and HCQ

Once trapped in acidic organelles, CQ and HCQ can only be extruded by exocytosis and/or through the multidrug resistance protein p-glycoprotein (Yamagishi et al., 2013; Zhitomirsky and Assaraf, 2016, 2017). Given their ability to de-acidify acidic organelles and acidify cytosolic pH, CQ and HCQ may disturb many key aspects of cell biology such as organellar biology and inter-organellar signaling. However, decades of clinical usage have shown that CQ and HCQ are relatively safe drugs. Especially, CQ and HCQ have been used during pregnancy in patients with autoimmune disorders, and recent systematic reviews and meta-analyses suggest that maternal use of HCQ during pregnancy does not increase risk of major congenital malformations (Gotoff, 1968). The reported tissue side effects of CQ and HCQ, such as retinopathy (Marmor et al., 2016; Nguyen et al., 2018), cardiomyopathy (Yogasundaram et al., 2018), neuromyotoxicity (Estes et al., 1987), and harmful effects on sperm quality (Leroy et al., 2015), are likely due to abnormal accumulation of these drugs in tissues with chronic use at a higher dosage (Adelusi and Salako, 1982; Chandler et al., 2020).

## CQ and HCQ as Adjuvant Cancer Therapy in Clinical Trials

Preclinical studies support of the use of CQ and HCQ in anti-cancer therapy, especially in combination with chemotherapeutic drugs because they are able to sensitize tumor cells to a variety of chemotherapeutic drugs and enhance their efficacy in tumor cell killing. Currently, clinical trials are evaluating the activity of CQ and HCQ in different cancer types and in combination with various standard treatments. Many of these clinical studies are designed to assess their anti-cancer effects as inhibitors of autophagic flux. Findings from these clinical studies are in favor of the repurposing of CQ and HCQ as adjuvant cancer therapies (Sotelo et al., 2006; Barnard et al., 2014). A meta-analysis of clinical trials (Xu et al., 2018) indicates that CQ and HCQ, as autophagy-inhibitor-based therapy enhances a better treatment response and outcome when compared to chemotherapy or radiation therapy alone without inhibiting autophagy. Although many clinical trials are underway to further evaluate the effectiveness of CQ and HCQ used in combination with chemotherapeutic drugs, a number of trials have been completed and show beneficial effects in different cancer types (Table 1). One clinical trial for patients with glioblastoma, which has a poor-prognosis of survival, found that patients treated with CQ in combination with chemotherapy and radiation had a significantly longer survival (Briceño et al., 2003). Another clinical study examined the effects of HCQ on patients with pancreatic adenocarcinoma as adjuvant therapy to gemcitabine. In this study, patients who had more than a 51% increase in the autophagy marker LC3-II in their circulating peripheral blood mononuclear cells showed improvement in disease-free survival and in overall survival (Boone et al., 2015). One other clinical study found that treatment with CQ plus whole-brain irradiation improved the control of metastases in the brain; however, in this particular study CQ did not improve the response rate or the overall survival (Rojas-Puentes et al., 2013). The anti-cancer effects of CQ and HCQ being tested in different cancer types at different stages, and in combination with various chemotherapeutic treatment, may contribute to the inconsistencies in clinical findings. It should be noted that in addition to lysosome de-acidification and inhibition of autophagic flux, CQ and HCQ affect many other aspects of the tumor microenvironment. Thus, larger and more definitive studies of CQ and HCQ as adjuvant cancer therapy are warranted (Verbaanderd et al., 2017).

**TABLE 1:** A brief list of completed clinical trials on the efficacy of CQ and HCQ in combination with chemotherapeutic drugs.

Treatment	Tumor	Potential pH-dependent mode of action	Result	References
CQ + Carmustinevs. Carmustine	Glioblastoma	Inhibits autophagy flux	Median Survival (mo.) 33 (±5) vs. 11 (± 2)	Briceño et al., 2003
CQ + Carmustine vs. Carmustine	Glioblastoma	Inhibits autophagy flux	Median Survival (mo.) 24 vs. 11	Sotelo et al., 2006
CQ + Irradiation vs. Irradiation	Brain Metastasis	Inhibits autophagy flux	CQ increased PFS time by 28.8%	Rojas-Puentes et al., 2013
HCQ + Gemcitabine	Pancreatic Adenocarcinoma	Inhibits autophagy flux	35 vs. 11 mo.*	Boone et al., 2015
HCQ + Doxorubicin vs. Doxorubicin	Non-Hodgkin Lymphoma	Inhibits autophagy flux	93.3% vs. 60% ORR	Barnard et al., 2014

*Patients who had more than a 51% increase in the autophagy marker LC3-II in circulating peripheral blood mononuclear cells was 35 months vs. who did not at 11 months. ORR, Overall Response Rate; PFS, Progression Free Survival.

## Proton Pump Inhibitors

Proton pump inhibitors (PPI), with varying efficacies, enhance the effectiveness of chemotherapeutic agents against tumor growth and metastasis (Fais, 2010, 2015; Taylor et al., 2015; Lugini et al., 2016; Spugnini and Fais, 2017). Similar to CQ and HCQ, PPIs alter the acidity of the tumor microenvironment, which induces cytotoxicity of tumor cells, reverses drug resistance, and reduces cancer metastasis (Walsh et al., 2015). PPIs increase the extracellular pH and acidify the cytosol; as such PPIs can direct chemotherapy agents such as doxorubicin to the nucleus (Luciani et al., 2004; Schwartz et al., 2017). In vivo studies demonstrate that PPIs, such as omeprazole and lansoprazole, enhance tumor sensitivity to cisplatin and paclitaxel (Luciani et al., 2004; Azzarito et al., 2015). The significance of pH effects in tumor cell homeostasis has been illustrated with PPI single treatments and in combination therapy, which both inhibited tumor proliferation and had a dose-dependent apoptotic-like cytotoxicity (De Milito et al., 2007, 2010; Canitano et al., 2016; Federici et al., 2016; Iessi et al., 2017; Lugini et al., 2017). The importance of pH alteration in cancer therapeutics is also highlighted when studies utilized a seemingly simple alkaline supplementation alone in murine melanoma and prostate adenocarcinoma that inhibited tumor cell growth and progression (Azzarito et al., 2016; Astigiano et al., 2017). Furthermore, exosome trafficking such as cellular-content release and cell-to-cell transmission is a well-known delivery mechanism in tumor malignancy. Pretreatment with PPIs or even buffering the acidic medium led to an inhibition of exosome uptake and release by a variety of cancer types including melanoma cells, colon, breast, prostate, and osteosarcoma (Parolini et al., 2009; Federici et al., 2014; Logozzi et al., 2018).

To further elucidate the role of altering acidity by PPIs in tumor therapy, a study illustrated that inhibiting ATP6L (a subunit of v-ATPase that regulates acidic endolysosomes) or TM9SF4 (a v-ATPase associated protein) with small interfering RNA enhanced the effectiveness of chemotherapy agents in cancer cell lines (You et al., 2009; Lozupone et al., 2015). In addition, autophagy as a survival mechanism to drug-induced cytotoxicity is illustrated by the PPI esomeprazole, which induces autophagy; but inhibition of autophagy increases the cytotoxicity of esomeprazole (Marino et al., 2010, 2012). Furthermore, PPIs increase tumor extracellular microenvironment pH and correct T-cell dysfunction as well as improve T-cell-based treatment (Calcinotto et al., 2012). Lastly, PPIs are effective in reversing tumor resistance when used in combination with chemotherapy vs. chemotherapy alone in companion animals and clinical studies (Spugnini et al., 2011, 2014; Ferrari et al., 2013; Walsh et al., 2015; Wang et al., 2015; Falcone et al., 2016; Marchetti et al., 2016). These findings further emphasize the significance of pH in tumors and the therapeutic impact of proton and metabolic regulation in cancer treatment (Gillies et al., 2019; Pillai et al., 2019).

## Summary

The altered tumor microenvironment plays an important role in the development of tumors, their response to therapy, and the development of chemoresistance. As diprotic weak bases, CQ and HCQ not only affect the luminal pH of acidic organelles (along the endocytic pathway and biosynthetic secretory pathway) but also affect cytosolic pH. Such CQ-and HCQ-induced pH alterations in various cellular compartments, in a large part, contribute to their ability to overcome chemoresistance. Thus, repurposing FDA-approved CQ and HCQ as adjuvant cancer therapy to overcome chemoresistance is promising. The full spectrum of their effects on pH changes and tumor microenvironments suggests that larger and more definitive clinical studies of CQ and HCQ as adjuvant cancer therapies are warranted.

## Author Contributions

PH and XC drafted the manuscript. PH prepared the figure. XC and JG edited the manuscript. All authors contributed to the article and approved the submitted version.

## Conflict of Interest

The authors declare that the research was conducted in the absence of any commercial or financial relationships that could be construed as a potential conflict of interest.
